# *catena*-Poly[[tetra­aqua­manganese(II)]-μ-5-carboxyl­ato-1-carboxyl­atomethyl-2-oxidopyridinium-κ^2^ *O* ^5^:*O* ^1^]

**DOI:** 10.1107/S1600536811025967

**Published:** 2011-07-06

**Authors:** Hong-Yan Yuan, Mei-Xiang Jiang, Yun-Long Feng

**Affiliations:** a Zhejiang Key Laboratory for Reactive Chemistry on Solid Surfaces, Institute of Physical Chemistry, Zhejiang Normal University, Jinhua, Zhejiang 321004, People’s Republic of China

## Abstract

In the title coordination polymer, [Mn(C_8_H_5_NO_5_)(H_2_O)_4_]_*n*_, the Mn^II^ atom is coordinated by two carboxyl­ate O atoms from two 5-carboxyl­ato-1-carboxyl­atomethyl-2-oxidopyridinium (*L* ^2−^) ligands and by four water mol­ecules in a distorted octa­hedral geometry. The *L* ^2−^ ligands bridge the Mn atoms into an infinite chain motif along [100]; the chains are further inter­linked by O—H⋯O hydrogen bonds into a three-dimensional supra­molecular net.

## Related literature

For the use of ligands involving pyridyl and carboxyl­ate groups in the construction of novel complexes, see: Zhang *et al.* (2003 ▶); Jiang *et al.* (2010 ▶); Yang *et al.* (2010 ▶).
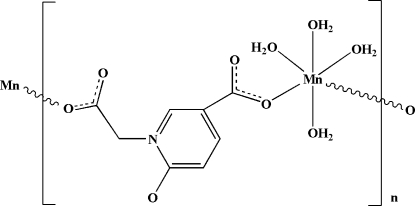


## Experimental

### 

#### Crystal data

[Mn(C_8_H_5_NO_5_)(H_2_O)_4_]
*M* *_r_* = 322.13
Monoclinic, 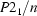

*a* = 5.1537 (2) Å
*b* = 21.2008 (9) Å
*c* = 10.9727 (4) Å
β = 99.182 (2)°
*V* = 1183.54 (8) Å^3^
*Z* = 4
Mo *K*α radiation
μ = 1.16 mm^−1^
*T* = 293 K
0.21 × 0.18 × 0.13 mm

#### Data collection

Bruker APEXII area-detector diffractometer
Absorption correction: multi-scan (*SADABS*; Sheldrick, 1996 ▶) *T*_min_ = 0.787, *T*_max_ = 0.858
11341 measured reflections
2680 independent reflections
2217 reflections with *I* > 2σ(*I*)
*R*_int_ = 0.075

#### Refinement

*R*[*F* ^2^ > 2σ(*F* ^2^)] = 0.029
*wR*(*F* ^2^) = 0.075
*S* = 1.03
2680 reflections
196 parameters
12 restraints
H atoms treated by a mixture of independent and constrained refinement
Δρ_max_ = 0.35 e Å^−3^
Δρ_min_ = −0.32 e Å^−3^

### 

Data collection: *APEX2* (Bruker, 2006 ▶); cell refinement: *SAINT* (Bruker, 2006 ▶); data reduction: *SAINT*; program(s) used to solve structure: *SHELXS97* (Sheldrick 2008 ▶); program(s) used to refine structure: *SHELXL97* (Sheldrick, 2008 ▶); molecular graphics: *SHELXTL* (Sheldrick, 2008 ▶); software used to prepare material for publication: *SHELXTL*.

## Supplementary Material

Crystal structure: contains datablock(s) I, global. DOI: 10.1107/S1600536811025967/ng5191sup1.cif

Structure factors: contains datablock(s) I. DOI: 10.1107/S1600536811025967/ng5191Isup2.hkl

Additional supplementary materials:  crystallographic information; 3D view; checkCIF report

## Figures and Tables

**Table 1:** Hydrogen-bond geometry (Å, °)

*D*—H⋯*A*	*D*—H	H⋯*A*	*D*⋯*A*	*D*—H⋯*A*
O1*W*—H1*WA*⋯O2^i^	0.86 (1)	1.83 (1)	2.6833 (18)	171 (2)
O1*W*—H1*WA*⋯O1^i^	0.86 (1)	2.67 (2)	3.307 (2)	132 (2)
O1*W*—H1*WB*⋯O5^ii^	0.84 (1)	2.43 (2)	3.071 (2)	134 (2)
O2*W*—H2*WA*⋯O3^iii^	0.84 (1)	1.88 (1)	2.7006 (19)	165 (2)
O2*W*—H2*WB*⋯O1^i^	0.84 (1)	2.01 (1)	2.8249 (18)	165 (2)
O3*W*—H3*WA*⋯O5^iv^	0.85 (1)	1.83 (1)	2.6733 (18)	176 (2)
O3*W*—H3*WB*⋯O2^ii^	0.85 (1)	1.94 (1)	2.7550 (18)	159 (2)
O4*W*—H4*WA*⋯O3*W*^v^	0.83 (1)	2.13 (1)	2.911 (2)	158 (2)
O4*W*—H4*WB*⋯O5	0.84 (1)	2.00 (1)	2.7270 (18)	145 (2)

Symmetry codes: (i) 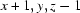; (ii) 
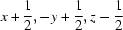
; (iii) 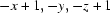; (iv) 
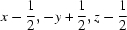
; (v) 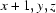.

**Fig. 1.**
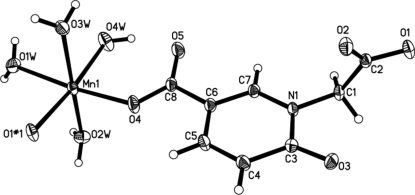
A view of the Mn(II) coordination environment in the structure of (I), showing the atom-labelling scheme. Displacement ellipsoids are drawn at the 30% probability level and H atoms are shown as small spheres of arbitrary radii. [Symmetry code: (1) x + 1,y,z - 1]

**Fig. 2.**
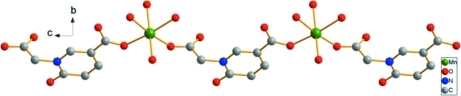
A ball-stick diagram showing the one-dimensional chain. All H atoms have been omitted for clarity.

**Fig. 3.**
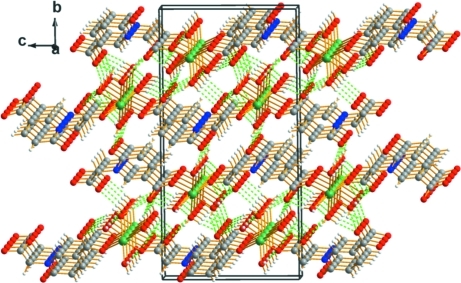
A packing structure of (I). The O—H···O interactions are depicted by green dashed lines.

## supplementary crystallographic information

### Comment

Versatile ligands involving pyridyl and carboxylate groups have been extensively employed to construct novel complexes owing to their various coordination modes (Zhang *et al.*, 2003; Jiang *et al.*, 2010; Yang *et al.*, 2010). Herein, we report the synthesis and crystal structure of a new complex, [MnL(H_2_O)_4_]_n_ (I).

In the title complex, the Mn(II) atom is six-coordinated by two carboxylic O atoms from two *L*^2-^ ligands and four water molecules, leading to a distorted octahedral environment (Fig. 1). Each *L*^2-^ ligand adopts a terminal monodentate bridging coordination mode to interconnect with the Mn(II) atoms forming a one-dimensional infinite polymeric chain motif. The shortest distance between the neighbour Mn(II) centers is 10.97 Å (Fig. 2). Additionally, with the aid of O—H···O hydrogen bonds found between the coordinated water molecules and carboxylato groups, the adjacent one-dimensional chains are further interlinked into a three-dimensional network (Fig. 3).

### Experimental

All the starting materials and solvents were obtained commercially and were used without further purification. H_2_L(0.197 g, 1.0 mmol), MnSO_4_.H_2_O (0.169 g, 1.0 mmol), Na_2_CO_3_ (0.106 g, 1.0 mmol) were mixed in 15 ml distilled water. Then the mixture was transferred into a Parr Teflon-lined stainless steel vessel (25 ml) and heated to 433 K for 72 h. Then, the reactor was cooled to room temperature at a speed of 5 degrees per hour. Colorless single crystals of title complex were obtained by slow evaporation of the filtrate over a few days.

### Refinement

The carbon-bound H-atoms were positioned geometrically and included in the refinement using a riding model [C—H 0.93, 0.97 Å *U*_iso_(H) = 1.2*U*_eq_(C)]. The oxygen-bound H-atoms was located in a difference Fourier maps and refined with the O—H distance restrained to 0.85 (2) Å [*U*_iso_(H) = 1.2*U*_eq_(O)].

### Crystal data

**Table:**

[Mn(C_8_H_5_NO_5_)(H_2_O)_4_]	*F*(000) = 660
*M**_r_* = 322.13	*D*_x_ = 1.808 Mg m^−^^3^
Monoclinic, *P*2_1_/*n*	Mo *K*α radiation, λ = 0.71073 Å
Hall symbol: -P 2yn	Cell parameters from 4396 reflections
*a* = 5.1537 (2) Å	θ = 2.1–27.4°
*b* = 21.2008 (9) Å	µ = 1.16 mm^−^^1^
*c* = 10.9727 (4) Å	*T* = 293 K
β = 99.182 (2)°	Block, colourless
*V* = 1183.54 (8) Å^3^	0.21 × 0.18 × 0.13 mm
*Z* = 4	

### Data collection

**Table:**

Bruker APEXII area-detector diffractometer	2680 independent reflections
Radiation source: fine-focus sealed tube	2217 reflections with *I* > 2σ(*I*)
graphite	*R*_int_ = 0.075
ω scans	θ_max_ = 27.4°, θ_min_ = 2.1°
Absorption correction: multi-scan (*SADABS*; Sheldrick, 1996)	*h* = −6→5
*T*_min_ = 0.787, *T*_max_ = 0.858	*k* = −27→27
11341 measured reflections	*l* = −14→14

### Refinement

**Table:**

Refinement on *F*^2^	Primary atom site location: structure-invariant direct methods
Least-squares matrix: full	Secondary atom site location: difference Fourier map
*R*[*F*^2^ > 2σ(*F*^2^)] = 0.029	Hydrogen site location: inferred from neighbouring sites
*wR*(*F*^2^) = 0.075	H atoms treated by a mixture of independent and constrained refinement
*S* = 1.03	*w* = 1/[σ^2^(*F*_o_^2^) + (0.0353*P*)^2^] where *P* = (*F*_o_^2^ + 2*F*_c_^2^)/3
2680 reflections	(Δ/σ)_max_ = 0.001
196 parameters	Δρ_max_ = 0.35 e Å^−^^3^
12 restraints	Δρ_min_ = −0.32 e Å^−^^3^

### Special details

**Table:**

Geometry. All e.s.d.'s (except the e.s.d. in the dihedral angle between two l.s. planes) are estimated using the full covariance matrix. The cell e.s.d.'s are taken into account individually in the estimation of e.s.d.'s in distances, angles and torsion angles; correlations between e.s.d.'s in cell parameters are only used when they are defined by crystal symmetry. An approximate (isotropic) treatment of cell e.s.d.'s is used for estimating e.s.d.'s involving l.s. planes.
Refinement. Refinement of *F*^2^ against ALL reflections. The weighted *R*-factor *wR* and goodness of fit *S* are based on *F*^2^, conventional *R*-factors *R* are based on *F*, with *F* set to zero for negative *F*^2^. The threshold expression of *F*^2^ > σ(*F*^2^) is used only for calculating *R*-factors(gt) *etc*. and is not relevant to the choice of reflections for refinement. *R*-factors based on *F*^2^ are statistically about twice as large as those based on *F*, and *R*- factors based on ALL data will be even larger.

### Fractional atomic coordinates and isotropic or equivalent isotropic displacement parameters (Å^2^)

**Table:**

	*x*	*y*	*z*	*U*_iso_*/*U*_eq_	
Mn1	0.82965 (5)	0.166986 (13)	0.24426 (2)	0.02401 (10)	
O1W	0.9758 (3)	0.21539 (7)	0.08980 (12)	0.0362 (3)	
H1WA	1.082 (4)	0.1969 (8)	0.0482 (18)	0.043*	
H1WB	1.035 (4)	0.2513 (6)	0.1101 (19)	0.043*	
O1	0.5110 (2)	0.12706 (7)	1.11849 (10)	0.0319 (3)	
O2	0.2636 (3)	0.15549 (6)	0.94224 (11)	0.0339 (3)	
O2W	1.0809 (3)	0.08634 (6)	0.22875 (13)	0.0349 (3)	
H2WA	1.016 (4)	0.0515 (7)	0.2020 (19)	0.042*	
H2WB	1.223 (3)	0.0930 (9)	0.2031 (19)	0.042*	
O3	0.2033 (3)	0.01801 (6)	0.84321 (11)	0.0344 (3)	
O3W	0.6083 (3)	0.25579 (6)	0.26185 (11)	0.0338 (3)	
H3WA	0.541 (4)	0.2760 (8)	0.1977 (11)	0.041*	
H3WB	0.695 (4)	0.2819 (7)	0.3121 (13)	0.041*	
O4W	1.1443 (3)	0.21688 (8)	0.36280 (12)	0.0450 (4)	
H4WA	1.298 (2)	0.2228 (12)	0.3515 (18)	0.054*	
H4WB	1.130 (4)	0.2130 (12)	0.4374 (11)	0.054*	
O4	0.6810 (3)	0.12960 (7)	0.39922 (10)	0.0380 (3)	
O5	0.8922 (3)	0.18553 (7)	0.55471 (11)	0.0458 (4)	
N1	0.5354 (3)	0.08088 (7)	0.80195 (11)	0.0235 (3)	
C1	0.6287 (4)	0.08637 (9)	0.93507 (14)	0.0259 (4)	
H1A	0.8038	0.1044	0.9478	0.031*	
H1B	0.6408	0.0445	0.9712	0.031*	
C2	0.4524 (3)	0.12667 (8)	1.00127 (14)	0.0234 (4)	
C3	0.3169 (4)	0.04332 (8)	0.76408 (15)	0.0255 (4)	
C4	0.2408 (4)	0.03795 (9)	0.63301 (16)	0.0329 (4)	
H4A	0.0989	0.0124	0.6020	0.039*	
C5	0.3701 (4)	0.06907 (9)	0.55330 (15)	0.0306 (4)	
H5A	0.3164	0.0644	0.4688	0.037*	
C6	0.5855 (3)	0.10852 (8)	0.59683 (14)	0.0247 (4)	
C7	0.6617 (3)	0.11259 (8)	0.72141 (14)	0.0246 (4)	
H7A	0.8047	0.1379	0.7521	0.029*	
C8	0.7325 (4)	0.14390 (8)	0.51148 (14)	0.0258 (4)	

### Atomic displacement parameters (Å^2^)

**Table:**

	*U*^11^	*U*^22^	*U*^33^	*U*^12^	*U*^13^	*U*^23^
Mn1	0.02227 (17)	0.03188 (17)	0.01840 (14)	−0.00270 (11)	0.00482 (10)	−0.00096 (10)
O1W	0.0419 (9)	0.0354 (7)	0.0353 (7)	−0.0012 (6)	0.0186 (6)	0.0025 (6)
O1	0.0263 (7)	0.0522 (8)	0.0173 (5)	−0.0076 (6)	0.0043 (5)	−0.0044 (5)
O2	0.0372 (8)	0.0405 (8)	0.0242 (6)	0.0071 (6)	0.0057 (5)	0.0040 (5)
O2W	0.0281 (7)	0.0323 (7)	0.0465 (8)	−0.0056 (6)	0.0126 (6)	−0.0034 (6)
O3	0.0410 (8)	0.0357 (7)	0.0280 (6)	−0.0135 (6)	0.0101 (6)	0.0031 (5)
O3W	0.0362 (8)	0.0342 (7)	0.0290 (6)	0.0054 (6)	−0.0006 (6)	−0.0050 (5)
O4W	0.0301 (8)	0.0731 (11)	0.0328 (7)	−0.0181 (8)	0.0082 (6)	−0.0135 (7)
O4	0.0428 (9)	0.0544 (9)	0.0186 (6)	−0.0152 (7)	0.0104 (5)	−0.0008 (6)
O5	0.0661 (11)	0.0483 (9)	0.0249 (6)	−0.0263 (8)	0.0134 (7)	−0.0039 (6)
N1	0.0253 (8)	0.0301 (8)	0.0154 (6)	−0.0033 (6)	0.0045 (5)	0.0001 (5)
C1	0.0252 (9)	0.0365 (10)	0.0157 (7)	−0.0036 (7)	0.0025 (6)	0.0026 (6)
C2	0.0234 (9)	0.0283 (9)	0.0188 (7)	−0.0087 (7)	0.0040 (6)	−0.0001 (6)
C3	0.0284 (10)	0.0255 (9)	0.0233 (8)	−0.0018 (7)	0.0059 (7)	0.0002 (6)
C4	0.0357 (11)	0.0380 (11)	0.0245 (8)	−0.0119 (9)	0.0037 (7)	−0.0043 (7)
C5	0.0340 (11)	0.0392 (10)	0.0183 (7)	−0.0023 (8)	0.0031 (7)	−0.0028 (7)
C6	0.0279 (10)	0.0274 (9)	0.0198 (7)	0.0021 (7)	0.0073 (7)	0.0009 (6)
C7	0.0242 (9)	0.0288 (9)	0.0218 (7)	−0.0032 (7)	0.0071 (7)	0.0004 (7)
C8	0.0304 (10)	0.0289 (9)	0.0194 (7)	0.0041 (8)	0.0072 (7)	0.0028 (7)

### Geometric parameters (Å, °)

**Table:**

Mn1—O4	2.1265 (12)	O4W—H4WB	0.837 (9)
Mn1—O1^i^	2.1435 (12)	O4—C8	1.255 (2)
Mn1—O2W	2.1676 (14)	O5—C8	1.248 (2)
Mn1—O4W	2.1833 (14)	N1—C7	1.357 (2)
Mn1—O1W	2.2136 (12)	N1—C3	1.387 (2)
Mn1—O3W	2.2258 (13)	N1—C1	1.468 (2)
O1W—H1WA	0.862 (9)	C1—C2	1.515 (2)
O1W—H1WB	0.838 (9)	C1—H1A	0.9700
O1—C2	1.2734 (18)	C1—H1B	0.9700
O1—Mn1^ii^	2.1435 (12)	C3—C4	1.434 (2)
O2—C2	1.240 (2)	C4—C5	1.353 (2)
O2W—H2WA	0.844 (9)	C4—H4A	0.9300
O2W—H2WB	0.837 (9)	C5—C6	1.411 (3)
O3—C3	1.2438 (19)	C5—H5A	0.9300
O3W—H3WA	0.849 (9)	C6—C7	1.363 (2)
O3W—H3WB	0.854 (9)	C6—C8	1.497 (2)
O4W—H4WA	0.830 (9)	C7—H7A	0.9300
			
O4—Mn1—O1^i^	91.77 (5)	C7—N1—C1	119.59 (14)
O4—Mn1—O2W	93.71 (5)	C3—N1—C1	117.71 (13)
O1^i^—Mn1—O2W	92.51 (5)	N1—C1—C2	113.31 (14)
O4—Mn1—O4W	91.85 (5)	N1—C1—H1A	108.9
O1^i^—Mn1—O4W	174.12 (6)	C2—C1—H1A	108.9
O2W—Mn1—O4W	91.88 (6)	N1—C1—H1B	108.9
O4—Mn1—O1W	174.26 (5)	C2—C1—H1B	108.9
O1^i^—Mn1—O1W	90.54 (5)	H1A—C1—H1B	107.7
O2W—Mn1—O1W	91.44 (5)	O2—C2—O1	124.35 (16)
O4W—Mn1—O1W	85.44 (5)	O2—C2—C1	120.54 (14)
O4—Mn1—O3W	89.48 (5)	O1—C2—C1	115.09 (15)
O1^i^—Mn1—O3W	92.25 (5)	O3—C3—N1	119.24 (15)
O2W—Mn1—O3W	174.18 (5)	O3—C3—C4	125.59 (17)
O4W—Mn1—O3W	83.15 (6)	N1—C3—C4	115.16 (14)
O1W—Mn1—O3W	85.18 (5)	C5—C4—C3	121.71 (17)
Mn1—O1W—H1WA	121.2 (14)	C5—C4—H4A	119.1
Mn1—O1W—H1WB	111.9 (14)	C3—C4—H4A	119.1
H1WA—O1W—H1WB	108.4 (14)	C4—C5—C6	120.82 (16)
C2—O1—Mn1^ii^	133.39 (11)	C4—C5—H5A	119.6
Mn1—O2W—H2WA	120.7 (14)	C6—C5—H5A	119.6
Mn1—O2W—H2WB	117.2 (14)	C7—C6—C5	117.54 (15)
H2WA—O2W—H2WB	110.4 (14)	C7—C6—C8	120.12 (16)
Mn1—O3W—H3WA	120.1 (13)	C5—C6—C8	122.32 (14)
Mn1—O3W—H3WB	112.5 (14)	N1—C7—C6	122.01 (16)
H3WA—O3W—H3WB	108.1 (13)	N1—C7—H7A	119.0
Mn1—O4W—H4WA	128.3 (15)	C6—C7—H7A	119.0
Mn1—O4W—H4WB	111.3 (15)	O5—C8—O4	124.56 (15)
H4WA—O4W—H4WB	113.2 (15)	O5—C8—C6	119.06 (14)
C8—O4—Mn1	130.38 (12)	O4—C8—C6	116.37 (16)
C7—N1—C3	122.70 (14)		
			
O1^i^—Mn1—O4—C8	−162.88 (17)	O3—C3—C4—C5	177.48 (19)
O2W—Mn1—O4—C8	104.49 (17)	N1—C3—C4—C5	−1.9 (3)
O4W—Mn1—O4—C8	12.48 (17)	C3—C4—C5—C6	−0.3 (3)
O1W—Mn1—O4—C8	−49.3 (6)	C4—C5—C6—C7	1.7 (3)
O3W—Mn1—O4—C8	−70.65 (17)	C4—C5—C6—C8	−179.72 (17)
C7—N1—C1—C2	106.33 (17)	C3—N1—C7—C6	−1.5 (3)
C3—N1—C1—C2	−73.06 (19)	C1—N1—C7—C6	179.13 (16)
Mn1^ii^—O1—C2—O2	−110.55 (19)	C5—C6—C7—N1	−0.8 (3)
Mn1^ii^—O1—C2—C1	70.8 (2)	C8—C6—C7—N1	−179.43 (15)
N1—C1—C2—O2	−5.9 (2)	Mn1—O4—C8—O5	−0.2 (3)
N1—C1—C2—O1	172.80 (14)	Mn1—O4—C8—C6	178.69 (12)
C7—N1—C3—O3	−176.60 (16)	C7—C6—C8—O5	−12.9 (3)
C1—N1—C3—O3	2.8 (2)	C5—C6—C8—O5	168.55 (18)
C7—N1—C3—C4	2.8 (2)	C7—C6—C8—O4	168.11 (17)
C1—N1—C3—C4	−177.83 (16)	C5—C6—C8—O4	−10.4 (3)

Symmetry codes: (i) *x*, *y*, *z*−1; (ii) *x*, *y*, *z*+1.

### Hydrogen-bond geometry (Å, °)

**Table:**

*D*—H···*A*	*D*—H	H···*A*	*D*···*A*	*D*—H···*A*
O1W—H1WA···O2^iii^	0.86 (1)	1.83 (1)	2.6833 (18)	171 (2)
O1W—H1WA···O1^iii^	0.86 (1)	2.67 (2)	3.307 (2)	132.(2)
O1W—H1WB···O5^iv^	0.84 (1)	2.43 (2)	3.071 (2)	134.(2)
O2W—H2WA···O3^v^	0.84 (1)	1.88 (1)	2.7006 (19)	165.(2)
O2W—H2WB···O1^iii^	0.84 (1)	2.01 (1)	2.8249 (18)	165 (2)
O3W—H3WA···O5^vi^	0.85 (1)	1.83 (1)	2.6733 (18)	176.(2)
O3W—H3WB···O2^iv^	0.85 (1)	1.94 (1)	2.7550 (18)	159 (2)
O4W—H4WA···O3W^vii^	0.83 (1)	2.13 (1)	2.911 (2)	158 (2)
O4W—H4WB···O5	0.84 (1)	2.00 (1)	2.7270 (18)	145 (2)

Symmetry codes: (iii) *x*+1, *y*, *z*−1; (iv) *x*+1/2, −*y*+1/2, *z*−1/2; (v) −*x*+1, −*y*, −*z*+1; (vi) *x*−1/2, −*y*+1/2, *z*−1/2; (vii) *x*+1, *y*, *z*.
